# A novel MVA-mediated pathway for isoprene production in engineered *E. coli*

**DOI:** 10.1186/s12896-016-0236-2

**Published:** 2016-01-20

**Authors:** Jianming Yang, Qingjuan Nie, Hui Liu, Mo Xian, Huizhou Liu

**Affiliations:** CAS Key Laboratory of Biobased Materials, Qingdao Institute of Bioenergy and Bioprocess Technology, Chinese Academy of Sciences, Qingdao, 266101 China; Key Lab of Applied Mycology, College of Life Sciences, Qingdao Agricultural University, Qingdao, 266109 China; Foreign Languages School, Qingdao Agricultural University, Qingdao, 266109 China

**Keywords:** Isoprene, MVA-mediated pathway, OleT_JE_, OhyA_EM_, *E. coli*

## Abstract

**Background:**

To deal with the increasingly severe energy crisis and environmental consequences, biofuels and biochemicals generated from renewable resources could serve as a promising alternative for replacing petroleum as a source of fuel and chemicals, among which isoprene (2-methyl-1,3-butadiene) in particular is of great significance in that it is an important platform chemical, which has been used in industrial production of synthetic rubber for tires and coatings or aviation fuel.

**Results:**

We firstly introduced fatty acid decarboxylase (OleT_JE_) from *Jeotgalicoccus* species into *E. coli* to directly convert MVA(mevalonate) into 3-methy-3-buten-1-ol. And then to transform 3-methy-3-buten-1-ol to isoprene, oleate hydratase (OhyA_EM_) from *Elizabethkingia meningoseptica* was overexpressed in *E. coli*. A novel biosynthetic pathway of isoprene in *E. coli* was established by co-expressing the heterologous *mvaE* gene encoding acetyl-CoA acetyltransferase/HMG-CoA reductase and *mvaS* gene encoding HMG-CoA synthase from *Enterococcus faecalis*, fatty acid decarboxylase (OleT_JE_) and oleate hydratase (OhyA_EM_). Furthermore, to enhance isoprene production, a further optimization of expression level of OleT_JE_, OhyA_EM_ was carried out by using different promoters and copy numbers of plasmids. Thereafter, the fermentation process was also optimized to improve the production of isoprene. The final engineered strain, YJM33, bearing the innovative biosynthetic pathway of isoprene, was found to produce isoprene up to 2.2 mg/L and 620 mg/L under flask and fed-batch fermentation conditions, respectively.

**Conclusions:**

In this study, by using metabolic engineering techniques, the novel MVA-mediated biosynthetic pathway of isoprene was successfully assembled in *E. coli* BL21(DE3) with the heterologous MVA upper pathway, OleT_JE_ from *Jeotgalicoccus* species and OhyA_EM_ from *Elizabethkingia meningoseptica*. Compared with traditional MVA pathway, the novel pathway is shortened by 3 steps. In addition, this is the first report on the reaction of converting MVA into 3-methy-3-buten-1-ol by fatty acid decarboxylase (OleT_JE_) from *Jeotgalicoccus* species. In brief, this study provided an alternative method for isoprene biosynthesis, which is largely different from the well-developed MEP pathway or MVA pathway.

**Electronic supplementary material:**

The online version of this article (doi:10.1186/s12896-016-0236-2) contains supplementary material, which is available to authorized users.

## Background

To address an increasingly severe energy crisis and its environmental consequences, biofuels and biochemicals generated from renewable resources, as a source of fossil fuels and chemicals, could serve as promising alternatives for petroleum [1, 2]. Isoprene becomes increasingly important as a vital platform chemical for the production of synthetic rubber [3] and aviation fuel [4].

So far, dimethylallyl diphosphate, the precursor of isoprene can be produced mainly through two different pathways, the methylerythritol 4-phosphate (MEP) pathway and mevalonate (MVA) pathway [5], which have already been adopted by different research groups for the biosynthesis of isoprene in *E. coli* [6–10]. The data show that MVA pathway is more effective in the isoprene production than the MEP pathway because of the regulatory mechanisms for the MEP pathway present in the native host [11].

Isoprene biosynthesis using the MVA pathway requires eight reactions that are catalyzed by seven or eight enzymes encoded by two operons. One operon consists of the genes *ERG10*, *ERG13* and *tHMGR* from *Saccharomyces cerevisiae* or *mvaE* and *mvaS* from *Enterococcus faecalis*. This operon catalyzes the formation of MVA from acetyl-CoA and is referred to as the ‘upper’ pathway. The other operon is composed of *ERG8*, *ERG12*, *ERG9*, and *IDI1*. This operon converts MVA to DMAPP and is called the ‘lower’ pathway. DMAPP is further converted to isoprene by isoprene synthase from *Populus alba* (IspS). Although much success has been achieved regarding isoprene biosynthesis, many problems remain to be solved, such as the intermediate imbalance resulting from heterologous over-expression of so many non-native genes in the host [12]. To overcome these hurdles, one approach is to employ a chromosome integration method to reduce the burden of cell growth resulting from the over-expression of heterologous genes [13, 14].

Recent advances in synthetic biology and metabolic engineering have made it possible to construct a new pathway to replace the native pathway by optimizing and assembling different sources of the enzymes. For example, Liao used an evolving citramalate synthase (CimA) from *Methanococcus jannaschii* to devise an innovative pathway that directly converted pyruvate to 2-ketobutyrate and avoided threonine biosynthesis. This constructed pathway is the simplest keto acid-mediated pathway for the biosynthesis of 1-propanol and 1-butanol generated from glucose [15]. Atsumi employed a strategy using the host’s highly active amino acid biosynthetic pathway to synhthesize 2-keto acid intermediates which were further transformed into higher alcohols by 2-keto-acid decarboxylases (KDCs) and alcohol dehydrogenases (ADHs) [16]. In this strategy, by adjusting the intermediates from amino acid biosynthesis pathways to alcohol production, biofuels were produced through two final unnatural steps.

**Fig. 1:**
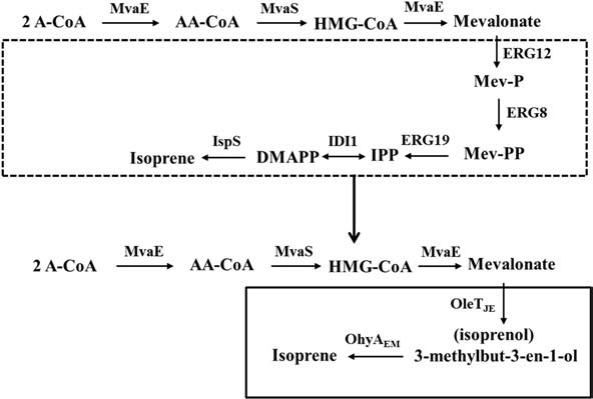
Production of isoprene via the novel MVA-mediated pathway used in this study. The dashed box shows the traditional pathway for MVA conversion to isoprene [8, 9], while the new pathway constructed in this study is within the solid-lined box. Enzyme symbols: MvaE (acetyl-CoA acetyltransferase/HMG-CoA reductase) and MvaS (HMG-CoA synthase) from *Enterococcus faecalis*; ERG12 (mevalonate kinase), ERG8 (phosphomevalonate kinase), ERG19 (mevalonate pyrophosphate decarboxylase) and IDI1 (IPP isomerase) from *Saccharomyces cerevisiae*; IspS (isoprene synthase) from *Populus alba*. OleT_JE_ from *Jeotgalicoccus* species; OhyA_EM_ from *Elizabethkingia meningoseptica*. Pathway intermediates: A-CoA, acetyl-CoA; AA-CoA, acetoacetyl-CoA; HMG-CoA, hydroxymethylglutaryl-CoA; Mev-P, mevalonate 5-phosphate; Mev-PP, mevalonate pyrophosphate. IPP, isopentenyl pyrophosphate; DMAPP, dimethylallyl pyrophosphate

In this paper, we designed an innovative biosynthetic route for isoprene production by assembling the MVA upper pathway from *Enterococcus faecalis*, the fatty acid decarboxylase (OleT_JE_) from *Jeotgalicoccus* sp. ATCC 8456 and the oleate hydratase (OhyA_EM_) from *Elizabethkingia meningoseptica* into a new pathway in *E. coli* (Fig. 1). This new pathway is of great importance for several reasons. First, fatty acid decarboxylase (OleT_JE_) was shown for the first time to catalyze the transition of MVA to 3-methyl-3-buten-1-ol. Furthermore, compared with the traditional MVA pathway, this novel pathway has been shortened by 3 steps. It represents the shortest MVA-mediated pathway for the production of isoprene from glucose. In summary, this paper develops a distinctive synthetic route to isoprene production that differs from the well-developed MEP pathway and MVA pathway by a large degree.

## Results and discussion

### Overexpression and functional analysis of OleT_JE_

**Fig. 2:**
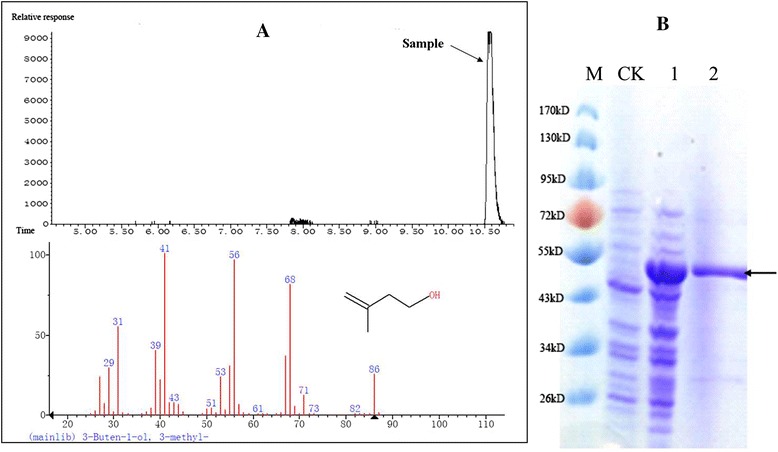
Enzymatic assay for 3-methyl-3-buten-1-ol production by OleT_JE_ using GC-MS and SDS-PAGE analysis. **a** GC-MS analysis of a 3-methyl-3-buten-1-ol sample produced by the OleT_JE_ assay mixtures. **b** SDS-PAGE analysis of OleT_JE_. CK: cell lysate from BL21(DE3) containing pCOLADuet-1. 1: crude cell extracts from YJM30. 2: purified OleT_JE_

The function of the fatty acid decarboxylase (OleT_JE_) from *Jeotgalicoccus* sp. ATCC 8456 to decarboxylate long-chain fatty acids into their corresponding terminal olefins has been previously demonstrated [17]. In this study, we determined whether OleT_JE_ could directly catalyze the MVA decarboxylation reaction. The nucleotide sequence of the fatty acid decarboxylase (OleT_JE_) generated from *Jeotgalicoccus* sp. ATCC 8456 was introduced into the plasmid pCOLADUet-1. The recombinant OleT_JE_ protein carrying a N-terminal six-histidine tag was purified from *E. coli*, and identified by SDS-PAGE (Fig. 2b). The enzyme activity was measured in a gas chromatography vial and a 3-methyl-3-buten-1-ol specific peak was detected by GC-MS (Fig. 2a). No detectable 3-methyl-3-buten-1-ol was formed when the purified enzyme or MVA was omitted from the assay. These results indicated that the isolated recombinant protein possessed MVA decarboxylase activity and was able to convert MVA to 3-methyl-3-buten-1-ol. To our knowledge, this reaction has not been previously documented.

In the native MVA pathway, MVA is phosphorylated twice and decarboxylated to form IPP. This process requires three enzymes, including mevalonate kinase, phosphomevalonate kinase and mevalonate diphosphate decarboxylase [18]. Then, using the enzyme pyrophosphatase or phosphatase, IPP can be converted into 3-methyl-3-buten-1-ol by removing the pyrophosphates [19]. To shorten the reaction steps of this pathway, we selected the enzyme fatty acid decarboxylase (OleT_JE_) from *Jeotgalicoccus* species, which has the ability to directly decarboxylate MVA into 3-methyl-3-buten-1-ol using only one step. The experimental results showed that the reaction catalyzed by the OleT_JE_ enzyme shortened the pathway and was able to convert MVA to 3-methyl-3-buten-1-ol in only one step without phosphorylation. To our knowledge, this is the first report of the above-mentioned reaction.

### Overexpression and functional analysis of OhyA_EM_

The capacity for cells containing oleate hydratase to transform oleic acid (OA) into 10-hydroxystearic acid (10-HSA) was first characterized by Wallen *et al*. in *Pseudomonas* sp. strain 3266 in 1962 [20]. Niehaus then showed that the reaction was reversible [21]. However, only in recent years was the gene encoding oleate hydratase in *Elizabethkingia meningoseptica* (formerly *Pseudomonas sp.*) cloned and expressed in *E. coli* [22]. Marliere demonstrated that oleate hydratase has the ability to catalyze the dehydration of isobutanol to form isobutene [23].

**Fig. 3:**
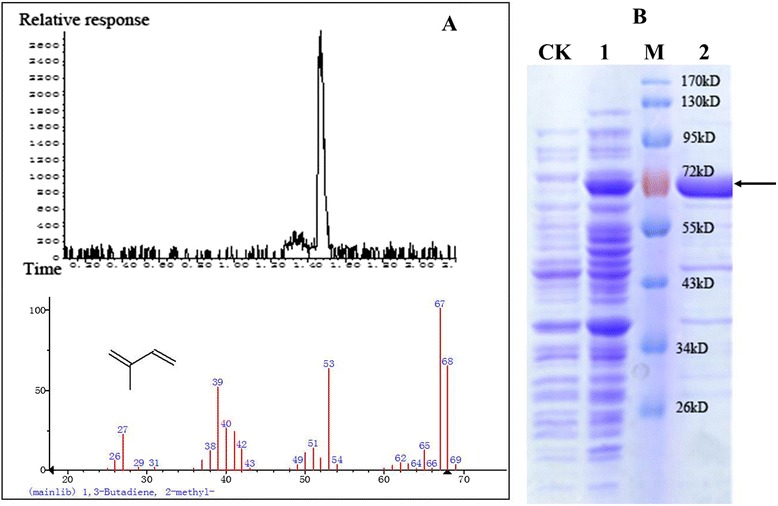
Enzymatic assay for isoprene production by OhyA_EM_ using GC-MS and SDS-PAGE analysis. **a** GC-MS analysis of a isoprene sample produced by the OhyA_EM_ assay mixtures. **b** SDS-PAGE analysis of OhyA_EM_. CK: cell lysate from BL21(DE3) containing pCOLADuet-1. 1: crude cell extracts from YJM31. 2: purified OhyA_EM_

According to the above-referenced studies, the enzyme oleate hydratase can dehydrate 3-methyl-3-buten-1-ol into isoprene. In our study, the nucleotide sequence of the *ohyA*_*EM*_ gene from *Elizabethkingia meningoseptica* was altered based on the preferred codon usage of *E. coli* and subsequently cloned into the vector pCOLADUet-1. The protein was expressed in *E. coli* BL21 (DE3) and purified using a nickel-affinity chromatography column. The band of the recombinant protein was observable on coomassie-stained SDS-PAGE gel of the crude cell extracts (Fig. 3b). The enzyme assay was conducted in a gas chromatography vial with GC-MS being used to verify an isoprene-specific peak (Fig. 3a). No detectable isoprene was produced when the purified enzyme or 3-methyl-3-buten-1-ol was omitted from the assay. The results suggested that the enzyme OhyA_EM_ from *Elizabethkingia meningoseptica* is capable of catalyzing the dehydroxylation of 3-methyl-3-buten-1-ol into isoprene.

### Establishing a novel biosynthetic pathway for isoprene in engineered *E. coli*

In previous experiments, the engineered strain YJM16 containing the efficient MVA upper pathway from *Enterococcus faecalis* was constructed, resulting in the accumulation of up to 1.31 g/L of MVA under flask culture conditions [9]. To subsequently obtain isoprene from glucose, we transformed the plasmid pYJM34 carrying the *oleT*_*JE*_ gene from *Jeotgalicoccus* species and *ohyA*_*EM*_ gene from *Elizabethkingia meningoseptica* into strain YJM16 harboring the MVA upper pathway. The resulting engineered strain YJM32 was inoculated in 50 ml fermentation medium and cultured at 37 °C which was further cultivated at 30 °C for 36 h with 0.5 mM IPTG addition into the broth when the OD_600_ attained about 0.6. The isoprene production by the strain YJM32 reached 17.6 μg/L. While the control engineered strain YJM35 only harboring upper MVA pathway and the fatty acid decarboxylase (OleT_JE_) cannot generate the isoprene. The results proved that a novel biosynthetic pathway for isoprene production containing the MVA upper pathway from *Enterococcus faecalis*, the *oleT*_*JE*_ gene from *Jeotgalicoccus* species and *ohyA*_*EM*_ gene from *Elizabethkingia meningoseptica* had been successfully constructed in *E. coli*.

In the previous studies, several research groups, including ours, established pathways for the conversion of MVA to isoprene. This process typically requires five reactions, including a two-step phosphorylation catalyzed by mevalonate kinase and phosphomevalonate kinase, a one-step decarboxylation catalyzed by mevalonate 5-diphosphate decarboxylase, a one-step isomerization catalyzed by IPP isomerase and a one-step dephosphorylation catalyzed by isoprene synthase [7, 9, 24]. This study is the first to use only two-step reactions to construct a new pathway for the conversion of MVA to isoprene by combining the *oleT*_*JE*_ gene from *Jeotgalicoccus* species and *ohyA*_*EM*_ gene from *Elizabethkingia meningoseptica*. Accordingly, from the starting acetyl-CoA to the final product isoprene, the entire pathway containing eight reactions was shortened to five reactions. The result is a promising step in the novel MVA-mediated biosynthetic pathway for isoprene production.

### Optimization of a biosynthetic pathway for isoprene production

**Fig. 4:**
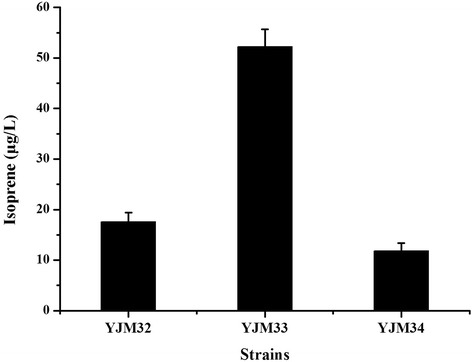
Optimization of the expression levels of *oleT*_*JE*_ and *ohyA*_*EM*_. The expression of *oleT*_*JE*_ aand *ohyA*_*EM*_ under the control of T7 promoter (YJM32) achieved much higher isoprene production than when the gene were under the control of the *ara*BAD promoter (YJM34). The strain YJM33 using a high copy number plasmid (pET-28a(+)) achieved the highest isoprene production (52.2 μg/L). The experiment was conducted in triplicate

To further enhance isoprene production, the expression levels of the *oleT*_*JE*_ gene from *Jeotgalicoccus* species and the *ohyA*_*EM*_ gene from *Elizabethkingia meningoseptica* were optimized by using different plasmid vectors containing different copy numbers and promoters. As is shown in Fig. 4, there achieved more isoprene production of the *oleT*_*JE*_ gene and *ohyA*_*EM*_ gene when under the control of the T7 promoter (YJM32) than that of the *ara*BAD promoter (YJM34). The highest isoprene production (52.2 μg/L) was found in the strain YJM33 harboring a high copy number plasmid, which was three-fold greater than the production of isoprene by YJM32 using lower copy number plasmids.

### Enhance isoprene production through optimizing the culture conditions

In our work, the “one-factor at-a-time” optimization strategy (Additional file 1: Fig. S1) was applied to augment isoprene productivity by optimizing the organic nitrogen source, induction temperature and IPTG concentration respectively. The results showed that the highest isoprene yield (2.2 mg/L) was obtained when the YJM33 strain was cultured in fermentation medium containing 20 g/L glucose as a carbon source, 9 g/L beef powder as an organic nitrogen source and induced with 0.25 mM IPTG at 31 °C whose combined optimization effect could contribute to an approximately 42-fold increase in isoprene production.

### Microbial isoprene production using the novel biosynthetic pathway

**Fig. 5:**
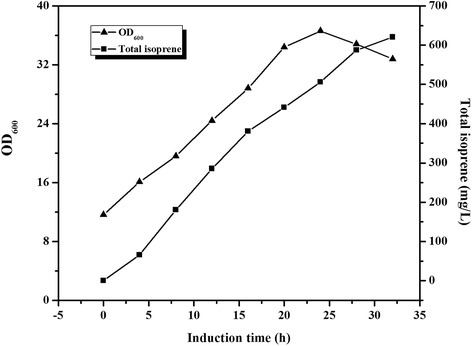
The time course of isoprene production by YJM33. Cell growth (▲) and isoprene accumulation (■) in YJM33. Cells were induced when the OD_600_ reached approximately 12 at 31 °C. Other experimental conditions are described in the section entitled “Fed-Batch Fermentation”

To assess the isoprene biosynthesis in a scalable process using the engineered strain YJM33 with the novel biosynthetic pathway encoded on the plasmids pYJM16 (pACY*-mvaE*-*mvaS*) and pYJM35 (pET28-*OleT*_*JE*_-*OhyA*_*EM*_), the fermentation of YJM33 under fed-batch condition was conducted on a 5-L scale. At an OD_600_ of ~12, 0.25 mM IPTG was put into the broth to induce the heterologous genes of the pathway for expression. After depleting the glucose initially present in the media, glucose solution (800 g l^−1^) was added to the cultures, and the residual glucose was restrained below 0.5 g/l to reduce acetate production. The OD_600_ at the end of the fermentation was ~36. As is shown in Fig. 5, isoprene gradually accumulated over the course of the fermentation and amounted to 620 mg/L with a productivity of 6.46 mg/L/h within 32 h (Fig. 5). In addition, isoprene production rose dramatically from 4 h to 16 h after being induced, and the productivity of isoprene attained 8.76 mg/L/h.

Although much progress has been made regarding the novel biosynthetic pathway of isoprene in *E. coli*, the present productivity remains too low and it is economically unfeasible for large scale production. The reason for the low yield could be the low catalytic activity of the enzymes OleT_JE_ and OhyA_EM_. Future studies should focus on enhancing the efficiency of the novel pathway using the following approaches: (1) The structures of both enzymes should be elucidated. Based on the structural data, it may be possible to enhance the catalytic efficiency by mutating key amino acids in the binding sites and catalytic active sites [25, 26] or to increase enzyme expression level by optimizing the Shine-Dalgarno sequence of enzyme [27]. (2) The pathway might be dynamically regulated using the dynamic sensor-regulator system (DSRS) developed by Keasling to bio-synthesize fatty acid-based products in *E. coli* [28]. The DSRS utilizes a transcription factor which can sense a crucial intermediate and dynamically regulate the expression level of genes related to target product synthesis. Consequently, if the natural sensor for crucial intermediate of MVA can be found, we can develop a DSRS for isoprene production to equilibrate metabolic pathway, thus enhancing product concentration, conversion efficiency and host’s genetic stability.

## Conclusions

In this paper, isoprene was synthesized through a distinctive biosynthetic pathway harboring the MVA upper pathway from *Enterococcus faecalis*, the *oleT*_*JE*_ gene from *Jeotgalicoccus* species and the *ohyA*_*EM*_ gene from *Elizabethkingia meningoseptica* in an engineered *E. coli* strain. The most optimized strain, YJM33, bearing the novel MVA-mediated biosynthetic pathway of isoprene, accumulated isoprene up to 2.2 mg/L and 620 mg/L under conditions of flask fermentation and fed-batch fermentation, respectively. Despite the relatively low level of isoprene production by this novel pathway, we have reduced the complexity of the native isoprene pathway by introducing two novel enzymes to catalyze the formation of isoprene from mevalonate in only 2 steps instead of 5.

To our knowledge, this is the first report of the conversion of MVA into 3-methyl-3-buten-1-ol by fatty acid decarboxylase (OleT_JE_) from *Jeotgalicoccus* species, and it is also the first to describe the catalysis of MVA to isoprene with simultaneous heterologous expression of the *oleT*_*JE*_ gene from *Jeotgalicoccus* species and the *ohyA*_*EM*_ gene from *Elizabethkingia meningoseptica*. Therefore, this study supplies an unusual synthetic route for bio-isoprene production that is very different from the well-characterized MEP pathway or MVA pathway.

## Methods

### Strains, plasmids and culture conditions

The strains and plasmids used in the present study are listed in Table 1. All of genes were expressed in *E. coli* BL21(DE3) to prepare the enzyme sample and biosynthesize the product. *E. coli* strains were grown in LB medium. For isoprene production, the different engineered strains were incubated in a shake-flask or under fed-batch fermentation conditions including defined medium consisted of 20 g/L glucose or 20 g/L glycerol, 0.3 g/L ferric ammonium citrate, 9.8 g/L K_2_HPO_4_, 9 g/L beef extract, 2.1 g/L citric acid monohydrate, 0.06 g/L MgSO_4_ and 1 ml of trace element solution which contained 0.29 g/L ZnSO_4_ · 7H_2_O, 0.37 g/L (NH_4_)_6_Mo_7_O_24_ · 4H_2_O, 0.25 g/L CuSO_4_ · 5H_2_O, 1.58 g/L MnCl_2_ · 4H_2_O and 2.47 g/L H_3_BO_4_. The strains fed on glycerol as a carbon source to produce isoprene when containing the plasmid of the *araBAD* promoter; strains without this plasmid were fed with glucose. Meanwhile, suitable antibiotics were added to the culture broth with the following concentrations: ampicillin (100 μg/ml), kanamycin (50 μg/ml), and chloramphenicol (34 μg/ml).

**Table 1:** Strains , plasmids used in this study

Name	Relevant characteristics	References
Strains		
* E.coli* BL21(DE3)	F^−^ *ompT hsd*S_B_ (r_B_^−^m_B_^−^) *gal dcm rne*131 λ(DE3)	Invitrogen
* E.coli* DH5α	*deoR*, recA1, endA1, hsdR17(rk-,mk+), phoA, supE44, λ-, thi-1, gyrA96, relA1	Takara
YJM30	*E.coli* BL21(DE3)/pYJM30	This study
YJM31	*E.coli* BL21(DE3)/pYJM32	This study
YJM32	*E.coli* BL21(DE3)/pYJM33,pYJM16	This study
YJM33	*E.coli* BL21(DE3)/pYJM34, pYJM16	This study
YJM34	*E.coli* BL21(DE3)/ pYJM35, pYJM16	This study
Plasmids		
pACYCDuet-1	P15A *ori*, *lacI* T7*lac*, Cm^r^	Novagen
pCOLADuet-1	ColA *ori*, *lacI* T7*lac*, Kan^r^	Novagen
pBAD 18	pBR322 *ori*, *ara*BAD, Amp^r^	[29]
pET-28a(+)	pBR322 *ori*, *lacI* T7*lac*, Kan^r^	Novagen
pYJM16	pACYCDuet-1 carrying *mvaE* and *mvaS* from *Enterococcus faecalis*	[9]
pYJM30	pCOLADuet-1 carrying *oleT*_*JE*_ from *Jeotgalicoccus* species	This study
pYJM31	pBAD18 carrying *oleT*_*JE*_ from *Jeotgalicoccus* species	This study
pYJM32	pCOLADuet-1 carrying *ohyA*_EM_ from *Elizabethkingia meningoseptica*	This study
pYJM33	pCOLADuet-1 carrying *oleT*_*JE*_ from *Jeotgalicoccus* species and *ohyA*_EM_ from *Elizabethkingia meningoseptica*	This study
pYJM34	pET-28a(+) carrying *oleT*_*JE*_ from *Jeotgalicoccus* species and *ohyA*_EM_ from *Elizabethkingia meningoseptica*	This study
pYJM35	pBAD18 carrying *oleT*_*JE*_ from *Jeotgalicoccus* species and *ohyA*_EM_ from *Elizabethkingia meningoseptica*	This study

### Plasmid construction

To improve the expression level of heterologous genes in *E. coli*, the nucleotide sequences of terminal olefin-forming fatty acid decarboxylase (OleT_JE_) gene (GenBank No. HQ709266.1) from *Jeotgalicoccus* sp. ATCC 8456 and the oleate hydratase (OhyA_EM_) gene (GenBank No. ACT54545.1) from *Elizabethkingia meningoseptica* were firstly evaluated assisted by online software (http://www.genscript.com/cgi-bin/tools/rare_codon_analysis) and then they were optimized according to the preferred codon usage of *E. coli* (http://www.jcat.de/). The optimized *oleT*_*JE*_ and *ohyA*_EM_ gene were chemically synthesized by Genray Company using plasmid pGH as the vector (called pGH-*oleT*_*JE*_ and pGH-*ohyA*_EM_, respectively). The *oleT*_*JE*_ gene fragment was obtained by digestion of pGH-*oleT*_*JE*_ with BamHI and SacI and was then ligated into the corresponding sites of pCOLADuet-1 to create pYJM30. Using the primers oleT-F (5′-CTAGCTAGCGGCAACACTTAAG AGGGATAAG-3′) and oleT-R (5′-CTAGGAGCTCTTATGTTCTGTCT ACAAC-3′) with plasmid pGH-*oleT*_*JE*_ as a template, the *oleT*_*JE*_ gene containing NheI and SacI sites was obtained by PCR. The isolated *oleT*_*JE*_ gene fragment was digested using NheI and SacI and inserted into the NheI/SacI sites of pBAD18 to yield plasmid pYJM31.

To construct the plasmid YJM32, the *ohyA*_EM_ gene was amplified by PCR using the primers ohya-F (5′-CGCGGATCCGAACCCGATCACCTCTAAATTCG-3′) and ohya-R (5′-CCCAAGCTTTTAACCACGGATACCTTTAACCCA-3′) with the plasmid pGH-*ohyA*_EM_ as a template. The isolated *ohyA*_EM_ gene fragment was excised with BamHI and HindIII and was inserted into the vector pCOLADuet-1 to form pYJM32. The *ohyA*_EM_ gene fragment was obtained by excision from pGH-*ohyA*_EM_ with BglII and XhoI and was introduced into pYJM30 to make pYJM33.

The *oleT*_*JE*_*-ohyA*_EM_ gene was digested from pYJM33 with BamHI and XhoI and ligated into pET-28a(+) to create pYJM34. Using the primers ohya-F1(5′-ACGCGTCGACGAACCCGATCACCTCTAAATTCG-3′) and ohya-R, the *ohyA*_EM_-1 fragment was amplified by PCR with plasmid pGH-*ohyA*_EM_ as a template. The amplified *ohyA*_EM_-1 gene was cut by SalI and HindIII and then introduced into the plasmid pYJM31 to create pYJM35.

The plasmids pYJM16 [9] and pBAD18 [29] were constructed as described previously.

### Purification of His_6_-OleT_JE_ and His_6_-OhyA_EM_ for *in vitro* enzyme assays

Approximately 0.5 ml of an overnight cultures inoculated into 50 ml of LB medium and cultivated at 37 °C. When the OD_600_ reached up to 0.9, 0.5 mM isopropyl β-D-thiogalactopyranoside (IPTG) was added into cultures to induce the protein production for 16 h at 28 °C. The cells were pelleted by centrifugation and resuspended in binding buffer (20 mM sodium phosphate, 0.5 M NaCl, pH 7.4). The cell membranes were destroyed using a sonic oscillator (Sonics VCX130). The lysate was centrifuged (12,000 g, 30 min), and the resulting supernatant was purified with a Ni-NTA purification system (Invitrogen) in which the protein concentration was determined using the Bradford Protein Assay Kit (Tiangen, China). The purification was appraised by SDS-PAGE.

### Enzymatic assay for 3-methyl-3-buten-1-ol production

The enzyme assay was performed as previously described [30]. 12 mM MgCl_2_, 5% glycerol, 50 mM Tris–HCl (pH 8.0), 250 mM ATP, 2 mM dithiothreitol (DTT), and 100 μM MVA were mixed and added into a gas chromatography vial containing 0.5 μM purified His_6_-OleT_JE_ enzyme. Enzyme-free or MVA-free control reactions were performed in parallel. Due to the volatility of 3-methyl-3-buten-1-ol, the headspace of the assay mixtures was sampled and characterized by GC-MS.

### GC-MS analysis of 3-methyl-3-buten-1-ol

The production of 3-methyl-3-buten-1-ol generated from the genetic strains was characterized by GC-MS analysis. The system was composed of a model 7890A network GC system (Agilent Technologies) and a model 5975C network mass selective detector (Agilent Technologies, Santa Clara, CA) in which A HP-INNOWAX capillary column (30 m × 0.25 mm × 0.25 μm, Agilent, Palo Alto, CA, USA) was adopted with helium as the carrier gas. The following temperature program was applied: 30 °C for 5 min, an increase of 7 °C/min to 100 °C, then rising from 100 °C to 250 °C at 25 °C/min. The injector temperature was set at 250 °C. A 1 μl sample was injected using a split injection mode with a split ratio of 20:1.

### Enzymatic assay for isoprene production

Purified His_6_-OhyA_EM_ was added to 20 mM Tris–HCl (pH 8.0) buffer containing 5 mM MgCl_2_, 50 mM NaCl, and 3-methyl-3-buten-1-ol with various amounts. Enzyme-free or substrate-free (3-methyl-3-buten-1-ol) control reactions were performed in parallel. The 1 ml reactions were placed in 2 ml gas-tight gas chromatography vials and was incubated at 37 °C for 72 h, and the headspace of the assay mixtures was sampled and measured by GC-MS.

### GC-MS analysis of isoprene

Putative isoprene products were detected by GC-MS. The GC-MS system was the same with the above described. The following temperature program was used: the initial temperature was maintained at 40 °C for 1 min, then increased to 70 °C at the constant flow rate of 4 °C/min, followed by an increase from 70 °C to 250 °C at the rate of 25 °C/min. The temperature of injector was set at 250 °C. A 1 μl sample was injected in the split injection mode with a split ratio of 7:1.

### Shake-flask cultures

Shake-flask experiments were carried out in triplicate in 600 ml sealed shake flasks consisted of 50 ml of fermentation medium as designed above. *E. coli* strains were inoculated into the medium and cultivated at 37 °C and 170 rpm. When the OD_600_ amounted to 0.6-0.9, 0.5 mM IPTG was added to the cultures and further cultivated at 30 °C for 24 h. Thereafter, a 1 ml headspace gas sample of the sealed cultures was extracted for test as was previously described [31]. Different concentrations of isoprene produced by the strains were calculated by means of converting the GC peak area to μg or mg of isoprene via a calibration curve. The standard curve was made as the follows: An isoprene standard (TCI-EP, Tokyo, Japan) of various concentrations was added in 600 ml sealed shake flasks consisted of 50 ml of fermentation medium. Due to the isoprene’s volatility, the headspace gas sample was extracted and detected using GC, the standard curve was made by various concentrations and their corresponding peak areas. Optimization of the fermentation medium as well as the process was given in Additional file 1.

### Fed-batch fermentation

The fed-batch fermentation of isoprene was conducted in a 5 L fermentor. The strain was grown in 2.5 L fermentation medium under the conditions of the fermentation temperature being maintained at 31 °C and the pH at 7.0 using NH_3_ · H_2_O. The flow rate of air was maintained at 1.3 VVM. 0.25 mM IPTG addition induced the cells at an OD_600_ of ~12 and the inducer was added every 8 h. During the whole course of fermentation, a glucose analyzer (SBA-40D, China) was adopted to measure the residual glucose which was kept below 0.5 g/L with glucose solution addition. In the meantime, the off-gas samples were taken from the fermentor and analyzed every 15 min by GC as described [31], and the growth of the bacterial culture was determined by measuring the OD_600_ with a spectrophotometer (Cary 50 UV–vis, Varian).

## Additional file

Additional file 1:
**Optimization of Fermentation Process and three Figs.** (DOCX 216 kb)

## Abbreviations

DMAPP: dimethylallyl pyrophosphate
IPP: isopentenyl pyrophosphate
MVA: mevalonate
MEP: methylerythritol 4-phosphate
IPTG: isopropyl β-D-thiogalactoside
PCR: polymerase chain reaction
