# Supplementary material for: A novel MVA-mediated pathway for isoprene production in engineered E. coli
Source: BMC Biotechnol. 2016 Jan 20;16:5. doi: 10.1186/s12896-016-0236-2 (PMC4719670; doi:10.1186/s12896-016-0236-2)

**Optimization of Fermentation Process**

Optimization of fermentation medium was carried out in shake-flask experiments in triplicate series of 50 ml of fermentation medium incubated with the strain YJM33. Cm (34 μg/mL) and Kan (50 μg/mL) were added when it was necessary. *E. coli* strains were cultured in the broth for initial production of isoprene and incubated in a gyratory shaker incubator at 37°C and 180 rpm. When the OD_600_ reached 0.6-0.9, IPTG was added to a final concentration of 0.25 mM, and the culture was further incubated at 30°C for 24 h. The isoprene extraction and analysis were performed as described previously.

**Effect of Organic** [**Nitrogen Source**](http://www.iciba.com/nitrogen_source)

The shake-flask cultures were incubated in initial medium with different organic [nitrogen source](http://www.iciba.com/nitrogen_source)s (5 g/L): beef extract (solarbio), beef powder (MDBio, Inc), tryptone

(Beijing AoBoXing Bio-Tech Co., Ltd) or yeast extract powder (Beijing AoBoXing Bio-Tech Co., Ltd)) at the above-mentioned culture conditions, and the isoprene products were detected.

**Effect of Induction Temperature**

The *E. coli* strain was inoculated in 50 ml of optimized fermentation medium and cultured at 37°C with shaking at 180 rpm. When the OD_600_ of the bacterial culture reached 0.6-0.9, the shake-flask cultures were incubated at different induction temperatures (25°C, 28°C, 31°C, 34°C or 37°C) for 24 h in 0.25 mM IPTG, and the isoprene products were assayed.

**Effect of IPTG Concentration**

The shake-flask culture was incubated in different inducer (IPTG) concentrations (0.05 mM, 0.1 mM, 0.25 mM, 0.5 mM or 1 mM) at the above-optimized temperature for 24 h, and the isoprene products were measured.

Fig. S1: **Effects of fermentation source and culture conditions on isoprene production by YJM33**. A: Effect of nitrogen sources on isoprene production; B: Effect of temperatures on isoprene production; C: Effect of the concentration of inducer on isoprene production. When OD_600_ reached 0.6-0.9, cultures were induced for 24 h using IPTG in shake-flasks. All the experiments were performed in triplicates. Optimized conditions: Nitrogen sources, beef power; Temperature, 31°C; IPTC concentration, 0.25 mM.


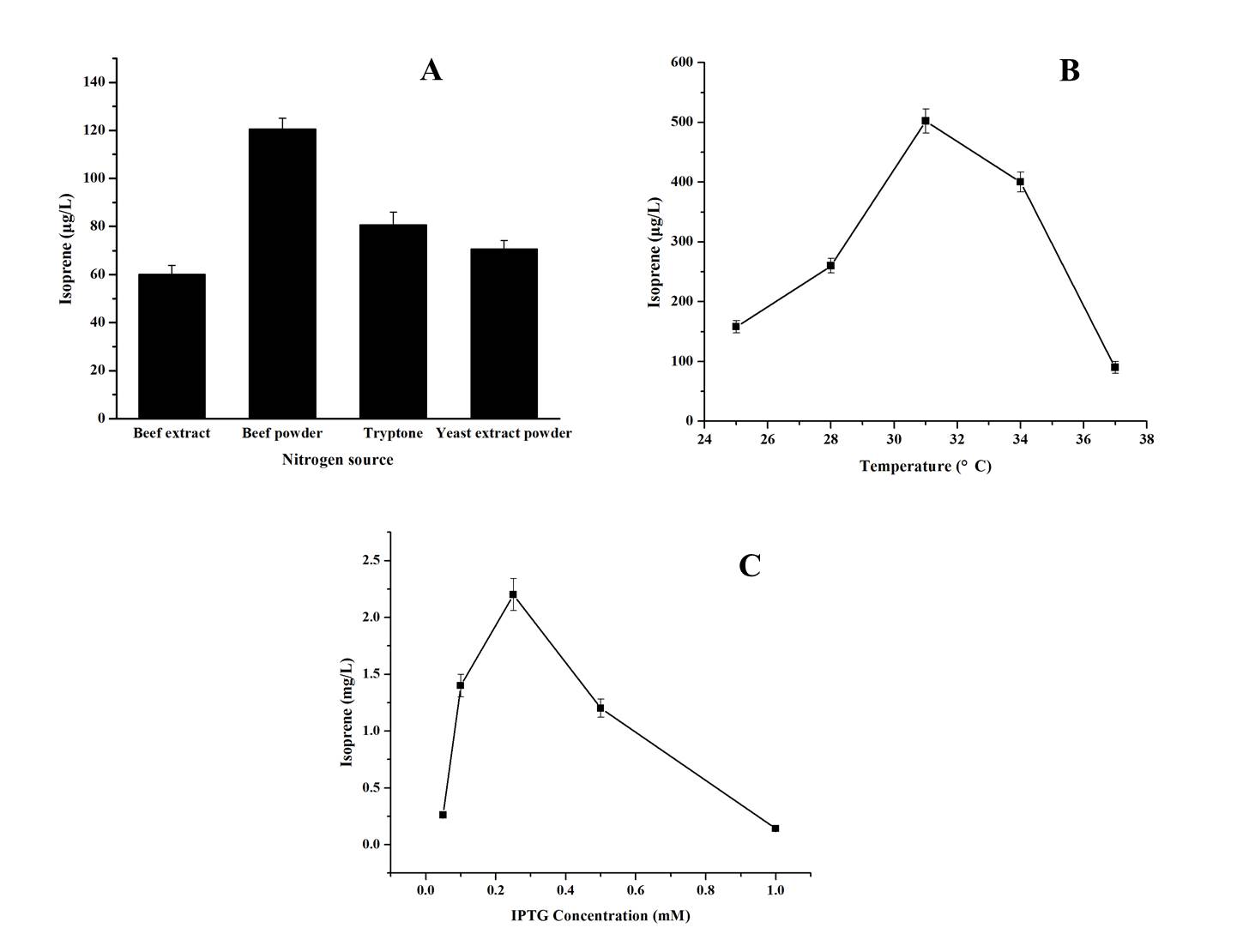


Fig. S2: **GC-MS analysis of** **3-methyl-3-buten-1-ol standard**.

A: Gas chromatography profile of a 3-methyl-3-buten-1-ol standard; B: Total ion mass spectral profile of a 3-methyl-3-buten-1-ol standard. The 3-methyl-3-buten-1-ol specific peak was indicated by arrow.


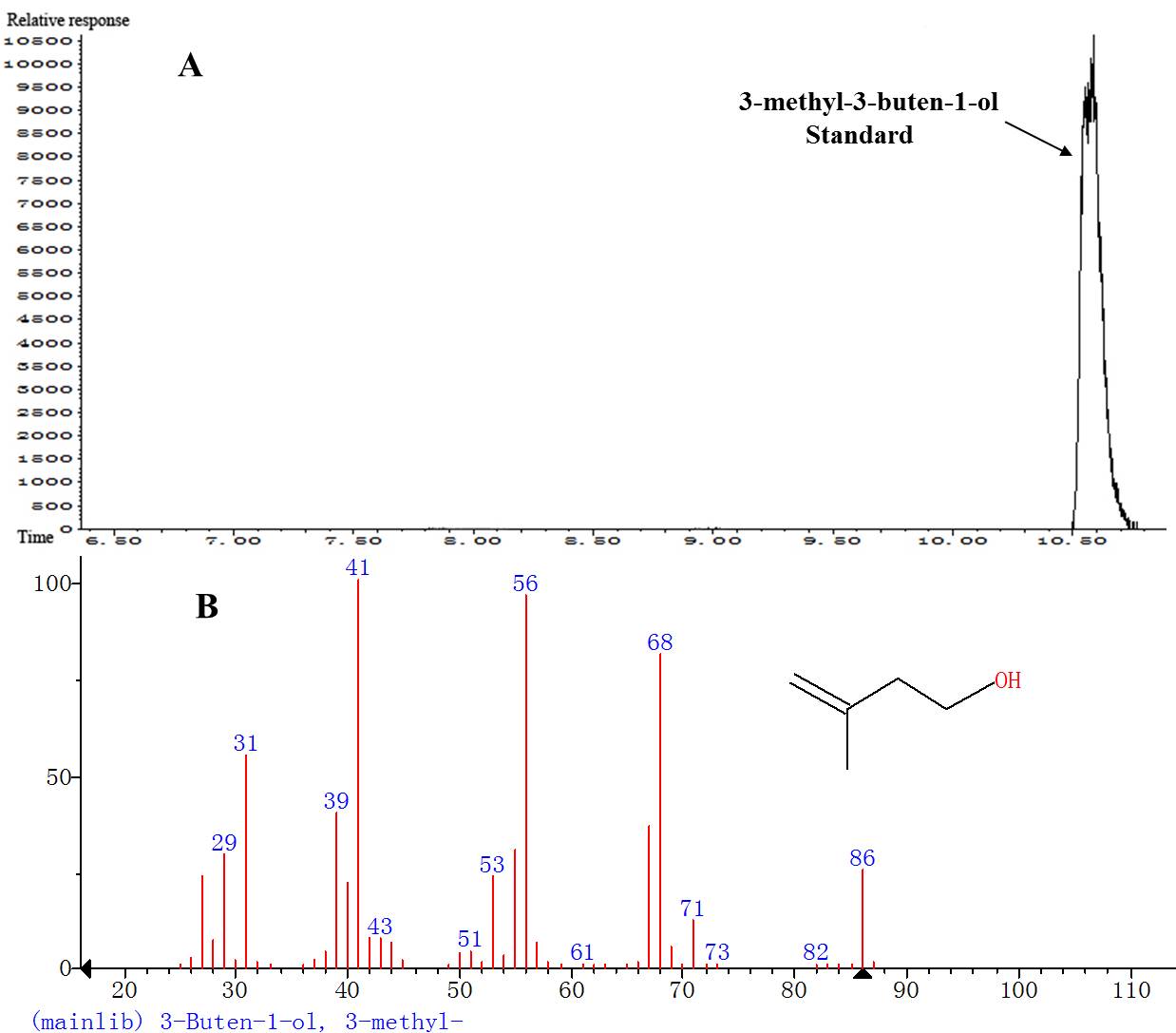


Fig. S3: **GC-MS analysis of isoprene standard**.

A: Gas chromatography profile of an isoprene standard; B: Total ion mass spectral profile of an isoprene standard. The isoprene specific peak was indicated by arrow.


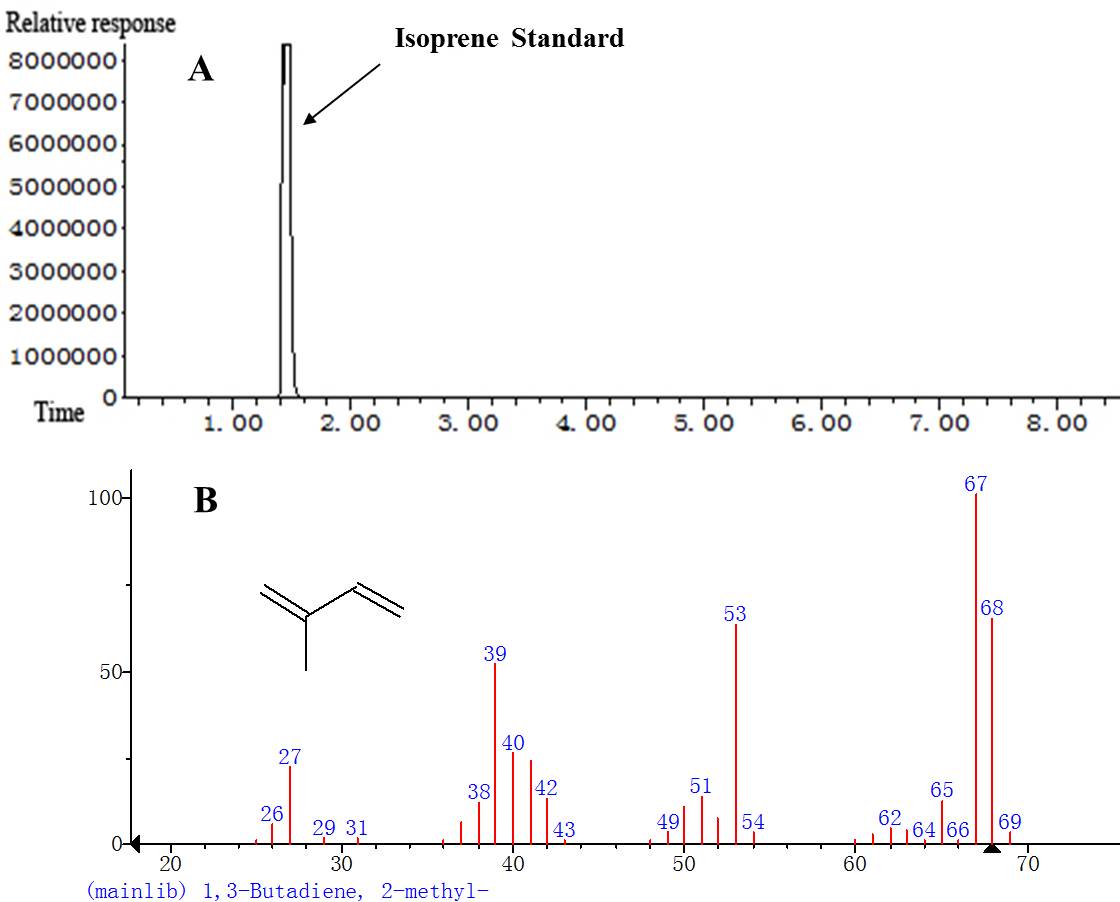

Supplement: Additional file 1: — Optimization of Fermentation Process and three Figs. (DOCX 216 kb) [file 12896_2016_236_MOESM1_ESM.docx]
